# A Computable Phenotype Algorithm for Postvaccination Myocarditis/Pericarditis Detection Using Real-World Data: Validation Study

**DOI:** 10.2196/54597

**Published:** 2024-11-25

**Authors:** Matthew Deady, Raymond Duncan, Matthew Sonesen, Renier Estiandan, Kelly Stimpert, Sylvia Cho, Jeffrey Beers, Brian Goodness, Lance Daniel Jones, Richard Forshee, Steven A Anderson, Hussein Ezzeldin

**Affiliations:** 1 IBM Consulting Bethesda, MD United States; 2 Departments of Enterprise Information Services and Pediatrics Cedars-Sinai Health System Los Angeles, CA United States; 3 Cedars-Sinai Health System Enterprise Information Services Los Angeles, CA United States; 4 DRT Strategies, Inc. Arlington, VA United States; 5 Center for Biologics Evaluation and Research Food and Drug Administration Silver Spring, MD United States

**Keywords:** adverse event, vaccine safety, interoperability, computable phenotype, postmarket surveillance system, fast healthcare interoperability resources, FHIR, real-world data, validation study, Food and Drug Administration, electronic health records, COVID-19 vaccine

## Abstract

**Background:**

Adverse events (AEs) associated with vaccination have traditionally been evaluated by epidemiological studies. More recently, they have gained attention due to the emergency use authorization of several COVID-19 vaccines. As part of its responsibility to conduct postmarket surveillance, the US Food and Drug Administration continues to monitor several AEs of interest to ensure the safety of vaccines, including those for COVID-19.

**Objective:**

This study is part of the Biologics Effectiveness and Safety Initiative, which aims to improve the US Food and Drug Administration’s postmarket surveillance capabilities while minimizing the burden of collecting clinical data on suspected postvaccination AEs. The objective of this study was to enhance active surveillance efforts through a pilot platform that can receive automatically reported AE cases through a health care data exchange.

**Methods:**

We detected cases by sharing and applying computable phenotype algorithms to real-world data in health care providers’ electronic health records databases. Using the fast healthcare interoperability resources standard for secure data transmission, we implemented a computable phenotype algorithm on a new health care system. The study focused on the algorithm's positive predictive value, validated through clinical records, assessing both the time required for implementation and the accuracy of AE detection.

**Results:**

The algorithm required 200-250 hours to implement and optimize. Of the 6,574,420 clinical encounters across 694,151 patients, 30 cases were identified as potential myocarditis/pericarditis. Of these, 26 cases were retrievable, and 24 underwent clinical validation. In total, 14 cases were confirmed as definite or probable myocarditis/pericarditis, yielding a positive predictive value of 58.3% (95% CI 37.3%-76.9%). These findings underscore the algorithm's capability for real-time detection of AEs, though they also highlight variability in performance across different health care systems.

**Conclusions:**

The study advocates for the ongoing refinement and application of distributed computable phenotype algorithms to enhance AE detection capabilities. These tools are crucial for comprehensive postmarket surveillance and improved vaccine safety monitoring. The outcomes suggest the need for further optimization to achieve more consistent results across diverse health care settings.

## Introduction

### Background

The US Food and Drug Administration (FDA) Center for Biologics Evaluation and Research (CBER) is responsible for ensuring the safety, purity, potency, and efficacy of biological products, including vaccines, allergenics, blood and blood products, and cells, tissues, and gene therapies [1]. In partnership with the Centers for Disease Control and Prevention, the FDA monitors vaccine safety through the vaccine adverse event reporting system (VAERS). Since the emergency use authorization of 4 COVID-19 vaccines—Pfizer-BioNTech, Moderna, Janssen, and Novavax—the FDA has been diligently analyzing adverse events (AEs) of special interest (AESIs) related to these vaccines and their boosters, including bivalent boosters.

VAERS receives spontaneous reports of suspected vaccine AEs from various sources, such as individuals, patients, clinical staff, and vaccine manufacturers. VAERS plays a vital role as an early warning system, relying on voluntary reports to monitor potential AEs. While this approach is invaluable, it can sometimes lead to underreporting, data quality issues, and challenges in establishing direct causality between vaccines and AEs. To enhance the robustness of monitoring, especially during public health emergencies, active surveillance efforts are essential in complementing and addressing the limitations of passive reporting systems like VAERS and FAERS. Established in 2017, CBER’s Biologics Effectiveness and Safety (BEST) Initiative creates a robust postmarket surveillance system for biologic products by leveraging real-world data (RWD) from sources such as electronic health records (EHRs) and claims data [2]. Through collaborations with various organizations, BEST enhances the detection and reporting of biologics-related AEs by assessing extensive health care data and scientific expertise [3,4].

The key focus of BEST is developing interoperable, computable phenotype algorithms that use standardized language to identify potential AEs in EHR databases [2]. This approach allows for automatic or semiautomatic detection and reporting of AEs with minimal effort from health care providers. In a study with the first site (site #1) [5], BEST successfully validated these algorithms, demonstrating their effectiveness in identifying AEs such as myocarditis (inflammation of the myocardium) and pericarditis (inflammation of the pericardium) following vaccination.

The ultimate goal is to implement a standardized, automated case reporting system across different EHR databases, enabling precise and timely identification of postvaccination AEs and their subsequent reporting to the FDA for further evaluation. This system aims to improve overall vaccine safety by swiftly identifying potential increases in AE incidence rates [4].

### Exchange Pilot Platform

To enhance CBER’s capability for postmarket surveillance, accessing a diverse and representative set of data on a near-national scale is essential. In a traditional federated network, including additional partners to achieve this scale, although essential, is prohibitively time-intensive. Therefore, a more efficient approach is needed to access data on a national scale and to address the potential undercounting of AEs.

To obtain the necessary RWD for potential AE cases from health care providers nationwide, we developed a pilot cloud-based platform to exchange EHR data with the FDA for analysis and active surveillance efforts. CBER BEST Innovative Methods initiative collaborated with the eHealth Exchange (eHX), a health information exchange, to explore the semiautomated detection and reporting of potential COVID-19 vaccine–related AEs. This platform serves as a valuable tool for public health awareness, safety, and transparency while minimizing the burden on health care providers in collecting clinical data.

**Figure 1:**
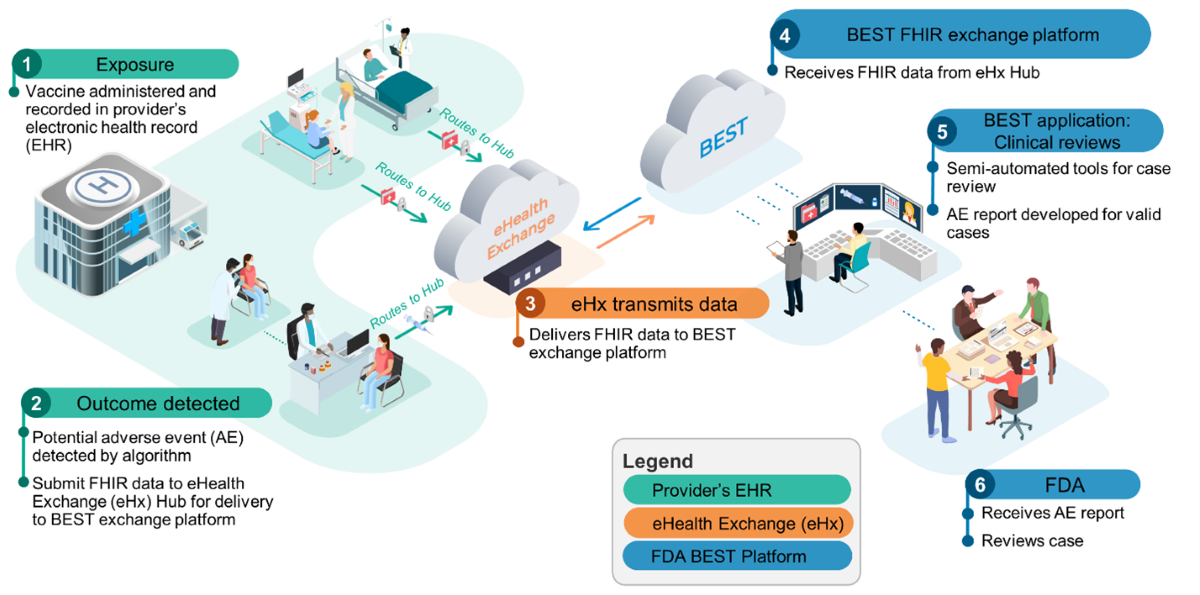
CBER (Center for Biologics Evaluation and Research) BEST (Biologics Effectiveness and Safety) Exchange pilot platform. FDA: US Food and Drug Administration; FHIR: Fast Healthcare Interoperability Resources.

Figure 1 demonstrates how data are exchanged from the health care provider through eHX to the FDA BEST platform.

This platform uses recent regulations under the 21st Century Cures Act Final Rule, which mandates that all clinical data be available through an application programming interface in the Fast Healthcare Interoperability Resources (FHIR) standard developed by Health Level Seven, Inc. (HL7) to improve interoperability between health systems by simplifying data exchange. These regulations facilitate seamless and secure access, exchange, and use of electronic health information [6,7]. FHIR is a standard developed by HL7 to facilitate interoperability between health systems. It is designed to improve upon existing standards by reducing implementation complexity without losing information integrity [3].

The EHR data provided through the exchanges give the FDA access to richer clinical datasets compared to those currently submitted to VAERS, reducing the need for further translation to a common data model by one or both parties. These data include unstructured clinician notes, which are often critical for AE analysis. This pilot platform is a first step in establishing an automated reporting system for AEs. To scale nationally, computable phenotypes must be dependable across health care provider systems, allowing the transmission of probable AE reports to the BEST platform for further evaluation to support postmarket safety and effectiveness.

This study models a nationwide automated reporting system by evaluating the effective distribution and implementation of an interoperable, computable phenotype algorithm at a new health care partner site (site #2), using a different EHR platform than at the initial validation site (site #1) [8]. The algorithm detected postvaccination AE case candidates for automatic reporting through our exchange pilot platform. The study focused on the algorithm’s implementation for identifying myocarditis and pericarditis, rare but significant risk factors following COVID-19 vaccinations, at site #2. Clinical data for detected cases were transmitted through the health care exchange and validated to assess detection and reporting accuracy. The results were compared to those from the previous validation study using the same phenotype algorithm at site #1.

A common issue with any distributed algorithm, especially for health care data, is the potential drop in performance when an algorithm developed at one site (site #1) is applied to a different site (site #2). Therefore, it is often difficult to generalize results from algorithms developed at multiple sites, leading to significant differences in positive predictive value (PPV) between sites, which can hinder the use of algorithms at a national level. This study aimed to determine whether the results of the algorithm were generalizable by comparing the PPV results from site #1 to those from site #2. The long-term goal is to accurately detect postvaccination AE cases by applying standardized phenotypes across different EHR databases.

Distributed algorithms require significant overhead due to the volume of translation to different data models or code systems. This study assessed the efficacy of the phenotype algorithm by measuring the time required for implementation by the health care organization. It also aimed to determine whether the BEST pilot platform received all necessary patient case data to validate the postvaccination AEs such as myocarditis/pericarditis. The evaluation criteria included the percentage of cases with insufficient evidence (eg, missing lab tests or clinical outputs needed for the case definition assessment) to complete a validation determination. This study is part of the FDA’s BEST initiative, and the findings from this validation study will help BEST staff to assess the long-term feasibility of scaling to a nationwide, automated detection using an active surveillance system.

## Methods

### Overview

The methods section is crucial for ensuring the accuracy and reliability of research findings. This study’s methodology encompasses the following aspects: computable phenotype development, phenotype distributed deployment, study period, data, and the process of reviewing medical records. Each methodological aspect plays a vital role in the comprehensive evaluation of our study's findings, ensuring the robustness and validity of the results.

### Computable Phenotype Development

For this validation study, we applied the algorithm developed for the postvaccination myocarditis/pericarditis phenotype used in the validation study by Holdefer et al [5] in 2023. The algorithm was built using a myocarditis/pericarditis case definition, which was current at the time of the Holdefer study. While a new case definition [9] was published in 2022, no changes to the developed phenotype algorithm were recommended. Therefore, we continued using the original algorithm for detection and the new case definition for our clinician validation. However, we made one change to the phenotype algorithm used in the Holdefer (2023) validation study by adding a vaccine exposure requirement. This requirement is for the myocarditis/pericarditis diagnosis to occur within a risk window of 0-42 days post vaccination.

Table S1 in the Multimedia Appendix describes the definitions of the terms. The specific search terms used to develop the code lists for myocarditis/pericarditis case definition concepts are listed in Tables S2-S6 in the Multimedia Appendix. Myocarditis/pericarditis were chosen due to reports of potential safety concerns after the initial distribution of COVID-19 vaccinations [9]. Recent research has indicated a reduction in the incidence of myocarditis/pericarditis associated with boosters compared to the initial 2-dose regimen for mRNA vaccines, likely due to interdose intervals [10,11]. However, myocarditis/pericarditis remain a monitoring concern given the recommendation for continued COVID-19 vaccinations.

### Phenotype Distributed Deployment

The BEST team provided a newly developed phenotype algorithm and code lists to our exchange data partner at site #2 in 2 standardized formats: clinical quality language (CQL) and observational medical outcomes partnership standard query language. The partner then implemented the algorithm in their Epic Clarity reporting databases. Detection of an AE triggered a secure submission of patient demographics to the FDA BEST platform.

The BEST team subsequently initiated a data request to the eHX, using demographics to locate patients via the FHIR search functionality. Future updates will enable the automatic transmission of relevant patient information through eHX to the BEST platform upon detecting potential AEs at the partner site, eliminating the need for manual intervention. To evaluate the algorithm's sharing and application success, we interviewed the IT team members responsible for the implementation and documented the findings, including the estimated hours needed for implementation and testing. These are described in the results section.

### Study Period

**Figure 2:**
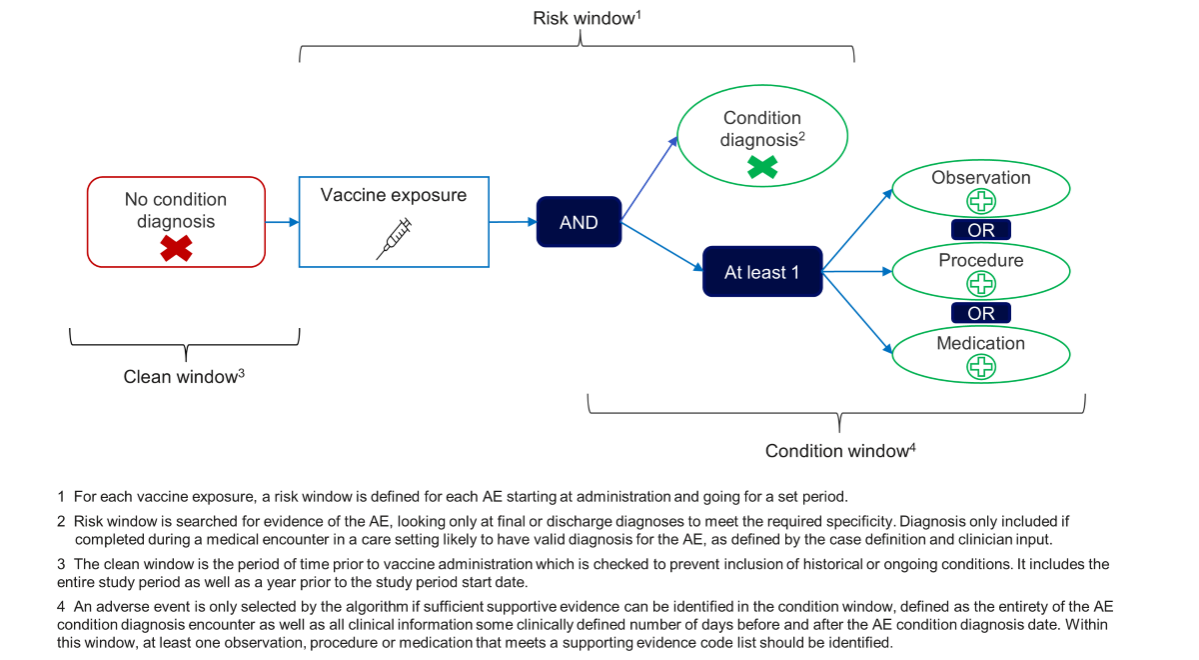
Standard algorithm template logic. AE: adverse event.

The study period was from December 14, 2020, when COVID-19 vaccinations received emergency use authorization, through April 28, 2023. The observation period began on December 14, 2019, to ensure all patients had at least 1 year of historical data, necessary to evaluate the clean window, as outlined in Figure 2.

### Data

The study population was derived from site #2, a health care provider partner recruited through their participation in the eHX system. eHX is a mature network of US exchange partners covering 77% of the nation’s state and regional health information exchanges, including the federal government and private health care networks. It securely shares clinical information over the internet using a standardized approach [12] and prenegotiated common agreements, providing timely access to RWD without the need for individual data use negotiations with partners. During the study period, site #2 provided care to 694,151 patients across over 6.5 million medical encounters. Table 1 presents the patient and encounter demographic breakdown for total and inpatient encounters, the only care settings considered for the postvaccination myocarditis/pericarditis phenotype.

Cases were selected using a distributed computable phenotype algorithm, which the site #2 team translated into a standard query language query for their Epic Clarity reporting database. The clinical data for the selected cases were provided to the BEST study team through site #2’s Epic FHIR application programming interface, which was queried by eHX and then sent to the BEST pilot platform. This included the data required for the algorithm, as well as additional data such as clinician notes, allergies, and procedures required for validation. A list of FHIR resources received for each patient identified by the algorithm is included in Table 2.

**Table 1:** Demographics of the academic health system for the study population (N=694,151).

Category		Inpatient		Total	
		Patients (n=78,946), n (%)	Encounters (114,254), n (%)	Patients (694,151), n (%)	Encounters (n=6,574,420), n (%)
**Age (years)**					
	<5	2134 (2.70)	2328 (2.04)	30,256 (4.36)	137,068 (2.08)
	5-17	888 (1.12)	1129 (0.99)	38,711 (5.58)	180,857 (2.75)
	18-24	2017 (2.55)	2699 (2.36)	45,859 (6.61)	222,254 (3.38)
	25-44	23,467 (29.73)	29,044 (25.42)	214,969 (30.97)	1,555,636 (23.66)
	45-64	17,727 (22.45)	26,886 (23.53)	194,912 (28.08)	2,057,258 (31.29)
	>65	32,677 (41.39)	52,131 (45.63)	167,988 (24.20)	2,418,968 (36.79)
	Missing	36 (0.05)	37 (0.03)	1456 (0.21)	2379 (0.04)
**Sex**					
	Male	45,327 (57.42)	63,534 (55.61)	317,393 (45.72)	2,808,578 (42.72)
	Female	33,590 (42.55)	50,691 (44.37)	375,258 (54.06)	3,762,854 (57.23)
	Missing or other	29 (0.04)	29 (0.03)	1500 (0.22)	2988 (0.05)
**Race**					
	White	51,531 (65.27)	74,462 (65.17)	420,483 (60.58)	4,231,991 (64.37)
	Black or African American	11,425 (14.47)	18,367 (16.08)	72,882 (10.50)	802,942 (12.21)
	Asian or Pacific Islander	6797 (8.61)	9247 (8.09)	62,467 (9.00)	630,886 (9.60)
	American Indian or Alaska Native	265 (0.34)	427 (0.37)	2104 (0.30)	25,755 (0.39)
	Other	7440 (9.42)	10,085 (8.83)	57,815 (8.33)	537,872 (8.18)
	Unknown	1171 (1.48)	1243 (1.09)	73,092 (10.53)	298,228 (4.54)
	Declined to answer	317 (0.40)	423 (0.37)	5308 (0.76)	46,746 (0.71)
**Ethnicity**					
	Hispanic	13,536 (17.15)	19,767 (17.30)	99,502 (14.33)	981,688 (14.93)
	Non-Hispanic	63,771 (80.78)	92,673 (81.11)	507,626 (73.13)	5,206,326 (79.19)
	Unknown	1639 (2.08)	1814 (1.59)	87,023 (12.54)	386,406 (5.88)

**Table 2:** Period of resources received for validation by resource type.

Resource	Date period
Allergy intolerance	Full clinical history
Condition	Full clinical history
Diagnostic report	Study period (12/14/2020-04/28/2023)
Document reference	Study period (12/14/2020-04/28/2023)
Encounter	Full clinical history
Immunization	Full clinical history
Location	All linked resources
Medication	All linked resources
Medication request	Full clinical history
Observation	Study period (12/14/2020-04/28/2023)
Patient	Single patient resource
Practitioner	All linked resources
Procedure	Full clinical history

### Process for the Review of Medical Records

All cases selected by the algorithm and pulled into the BEST platform were reviewed using a chart tool for BEST clinician review. Each case was assigned to 2 clinicians. The clinical validation used a patient’s comprehensive clinical history, including EHR data and all clinical notes. This dataset included information not used by the detection algorithm, such as different data types (eg, allergies, clinical notes, etc) and data that were filtered out (eg, admitting diagnosis, encounters with various care settings, etc). For each case, the reviewer evaluated whether the clinical data evidence met the specified case definition criteria. Relevant patient data for the case window were accessible and presented to the clinicians in a chart review application designed specifically for this purpose (see Figures S1-S7 in the Multimedia Appendix). Within the tool, clinicians could group items by type, search across all items and text, and request additional chart data to expand the window and access any available historical patient data.

All suspected AEs were validated using the conventional categories and definitions for rating diagnostic certainty of AEs: definite, probable, possible, or doubtful [13]. A case was classified as definite, probable, or possible if it met the published case definitions for that determination. Any case not meeting these criteria was labeled as doubtful. In cases of disagreement between the 2 clinicians, a third clinician made the final determination by reviewing the EHR data.

Clinicians could not confirm a case as positive (definite or probable) in case of insufficient information within the structured (data organized according to an explicit data model or data structure, often stored in a relational database) or unstructured (without a predefined data model, like free text) EHR data. In such instances, they marked the review as possible insufficient evidence, indicating that an AE might have occurred, but there was not enough documentation to meet the case definition requirements (eg, some case definitions may require specific test results that were not performed or not recorded in the EHR during patient care).

### Statistical Analysis

Statistical analysis in this study involved the examination and interpretation of data to evaluate the accuracy and reliability of our algorithm for identifying AEs. This section covers PPV, CIs, and interrater reliability. Each of these measures provides a distinct perspective on the algorithm's performance and the consistency of clinical adjudication.

### Positive Predictive Value

PPV was used to assess the accuracy of our algorithm. The PPV for each algorithm was determined as the proportion of positive AEs identified by the algorithm that were confirmed by clinical adjudication, out of all cases with sufficient evidence for clinical adjudication. This study’s PPV was compared to the one in the Holdefer validation study [5], which reported a PPV of 83.5% (95% CI 74.9%-89.6%).

Cases flagged as having insufficient evidence for validation were excluded from the calculation. These cases were initially considered as possible AEs due to some evidence of an AE. However, they lacked one or more pieces of crucial evidence required to meet the case definition (ie, no record of results for a lab test expected for use in diagnosis).

As indicated in Table 3, 4 cases labeled definite or probable were marked as true positive (where the clinician agreed with the algorithm’s prediction), while cases labeled possible or doubtful were marked as false positive (where the algorithm predicted a positive AE result, but clinicians could not confirm the occurrence of the AE).

**Table 3:** Case marking for PPV^a^ calculation.

Case labeling	Case marking
Insufficient evidence	Not included in PPV calculation
Definite or probable	True positives
Possible or doubtful	False positives

^a^PPV: Positive predictive value.

The PPV for this study was calculated as follows:

PPV=true positives (definite+probable)/all cases selected by algorithm with sufficient evidence (definite+probable+possible+doubtful).

As a sensitivity analysis, we also calculated PPV by including the cases without sufficient evidence as false positives. This alternative PPV was calculated as follows:

PPV=true positives (definite+probable)/all cases selected by algorithm (definite+probable+possible+doubtful).

### CIs

Since PPV is a binominal proportion, we calculated CIs for the performance metrics using the Agresti-Coull interval [14], the recommended method for estimating accurate CIs for binomial proportions such as PPV [15].

### Interrater Reliability

Interrater reliability was used to measure the extent to which the 2 physicians agreed in their AE assessment. It was calculated using Cohen kappa between the first 2 reviewers. Cohen κ measures the agreement between 2 raters classifying instances into mutually exclusive groups [16].

### Ethical Considerations

This study was conducted under the Food and Drug Administration's Sentinel initiative (Public Health Surveillance Activities), which are not deemed research per the guidance from the Office for Human Research Protections (OHRP), which has determined that the regulations they administer (45 CFR part 46) do not apply to the activities that are included in the FDA's Sentinel initiative.

## Results

### Overview

The results section presents the key findings of the study organized under Population Sample, Phenotype Implementation Results, and Overall PPV and Interrater Reliability Results. These result findings detail the selection of cases, the implementation of the phenotype algorithm, and the evaluation of the algorithm's performance and reliability.

### Population Sample

The computable phenotype algorithm selected cases from 114,254 inpatient encounters out of approximately 6.5 million total clinical encounters available during the study period. The algorithm identified 484 cases with a condition diagnosis and supporting evidence for myocarditis/pericarditis. Of those 484 cases, 30 met the algorithm’s additional vaccine exposure criteria, and 26 could be pulled into the BEST platform for our clinical validation study.

**Figure 3:**
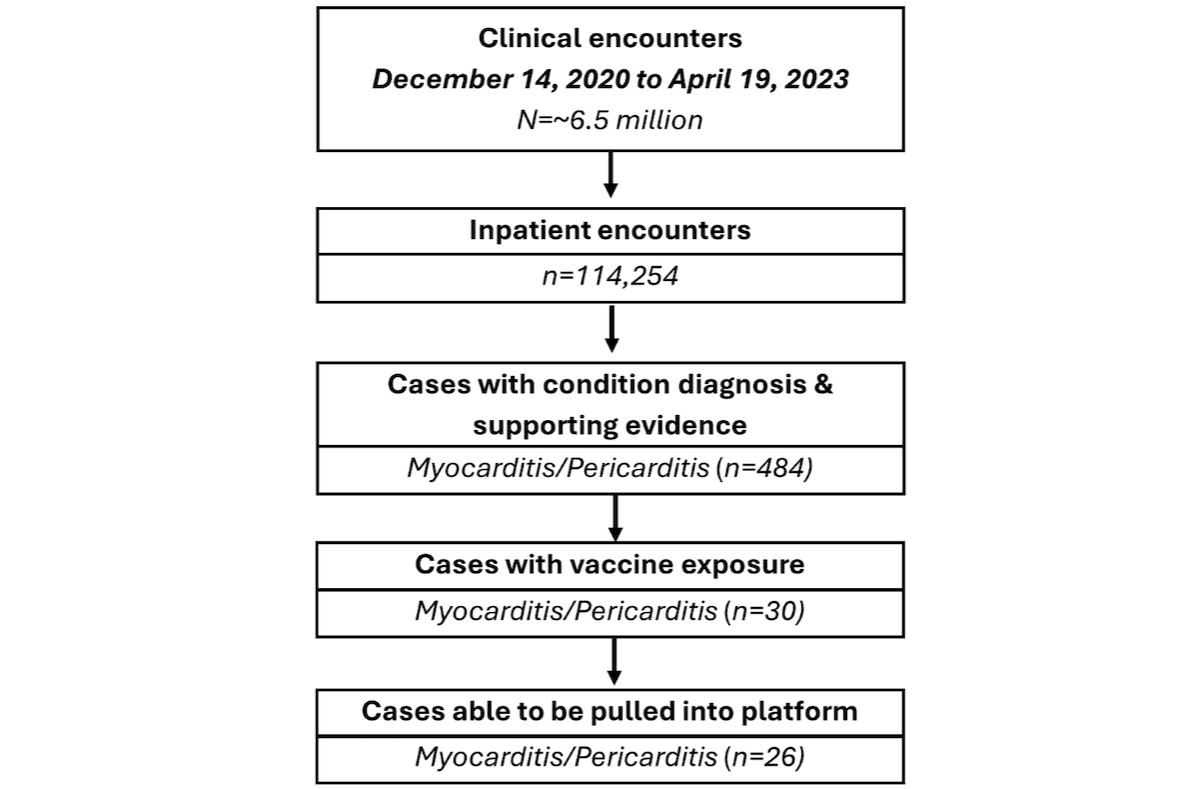
Study Population Consolidated Standards of Reporting Trials (CONSORT) diagram.

The cases that could not be retrieved through the eHX had demographic information matching multiple patients in the EHR database. This prevented data transfer for privacy reasons since the FDA cannot currently confirm that both patients had a potential postvaccination AE. A CONSORT (Consolidated Standards of Reporting Trials) diagram is included in Figure 3 to illustrate how the steps of the algorithm in filtering encounters produce our validation sample.

As indicated in Figure 3 cases were not pulled in because of multiple matches for the provided patient demographics.

### Phenotype Implementation Results

It took about 200-250 hours to successfully implement the phenotype on site #2’s Epic Clarity database. Considering the size of the EHR database, most of this time was spent designing an efficient data extraction solution and testing the logic to ensure it accounted for all possible scenarios, particularly in patients with multiple immunizations or AE diagnosis codes. The implementation team at site #2 believes that the query implementation time could be substantially reduced to approximately 20 hours from the current 200-250 hours at the next partner site using the same Epic EHR software by reusing the queries from this implementation. This is presented in further detail in the discussion section.

There were no issues matching the interoperable code list to the Epic Clarity database.

### Overall PPV and Interrater Reliability Results

The overall algorithm performance characteristics for the myocarditis/pericarditis computable phenotype as executed in the EHR system using cases with sufficient evidence are presented in Table 4.

For the 26 cases validated, PPV was 58.3% (95% CI 37.3%-76.9%), with approximately 7.7% of the data insufficient to make a determination. The interrater reliability Cohen κ score for the 26 reviewed cases was 0.699, which shows substantial agreement between the clinicians. This measure suggested sufficient reliability since the value was greater than 0.61, although it did not meet the higher threshold of 0.80 suggested by other articles [16].

**Table 4:** Site #2 total validation sample results.

Partner and final case classification (*Cedars Sinai*)	Detected cases
Definite, n	3
Probable, n	11
Possible, n	0
Doubtful, n	10
Total true positive cases (definite+probable), n	14
Total false positive cases (possible+doubtful), n	10
Total cases with sufficient evidence, n	24
PPV (definite+probable/total cases with sufficient evidence; %), % (95% CI)	58.3 (37.3-76.9)
Possible (insufficient evidence), n	2
Total cases, n	26
% of Cases with insufficient evidence, %	7.7
PPV–alternate calculation (definite+probable/total cases; %), % (95% CI)	53.8 (34.2-72.5)

## Discussion

### Overview

The discussion section highlights the findings of our study on the computable phenotype for postvaccination myocarditis/pericarditis AE across 2 health care organizations, site #1 and site #2. The discussion covers Principal Findings, Comparison of Validation to Previous Site Validation, and Methodological Considerations and Limitations. Our study underscores the importance of continued research to refine these algorithms and ensure their success in revolutionizing public health surveillance.

### Principal Findings

This study demonstrated that a simple, rules-based, postvaccination myocarditis/pericarditis AE-computable phenotype from one health care organization (site #1) [5] can be distributed and replicated at a separate health care organization (site #2) with comparable performance. However, the study also revealed a decline in PPV and a significant time cost for implementation at the new partner. The computable phenotype at site #2 had a PPV of 58.3% (95% CI 37.3%-76.9%), which was lower than that of 83.5% (95% CI 74.9%-89.6%) at site #1 [5]. This was driven largely by a higher percentage of cases with evidence of alternative diagnoses.

Additionally, the study showed that live, FHIR-formatted clinical data received through a health exchange-based platform are robust and interoperable enough for complex clinical case validation, in which we received direct data extracts from EHR tables from our health care partner from site #1 [5]. The proportion of cases identified by validating clinicians as containing insufficient data in this study was much lower than those in the previous study, where direct data extracts from EHR tables were used. Lastly, it demonstrated that our phenotypes could be implemented by health care analysts on EHR data unseen by algorithm developers, although with significant effort.

Feedback from our partner indicated that the phenotype took approximately 200-250 hours to implement. However, the implementation team believes that this time can be drastically reduced to approximately 20 hours by reusing the solution at subsequent data partners using the same Epic EHR software. They emphasized that the ease of deploying the algorithm is crucial for scaling the solution to other health organization data partners and provided several suggestions (as detailed in the section Methodological Considerations and Limitations) to make the process easier in the future.

### Comparison of Validation to Previous Site Validation

The PPV of this study’s validation was lower than the previously completed myocarditis/pericarditis validation (58.3%; 95% CI 37.3%-76.9%) versus (83.5%; 95% CI 74.9%-89.6%). Most false positives in this study occurred due to an alternative diagnosis that better explained the symptoms, such as myocardial infarction or pulmonary embolism [17]. This highlights the challenge of creating a reliable algorithm for complex diagnoses such as myocarditis or pericarditis, as they often present similarly to other conditions.

Possible explanations for the degradation in performance of the computable phenotype at site #2 include but are not limited to the following (Textbox 1).

If all cases with insufficient evidence were assumed negative, the PPVs of the 2 studies would be closer: 53.8% (95% CI 34.2%-72.5%) versus 63.7% (95% CI 55.2%-71.4%), and not statistically significant at *P*<.10.

**Possible explanations for the degradation in performance.**

- Different population compositions: The populations from different hospital systems with varying geographies and demographics, potentially affecting medical treatment types.
- Algorithm changes: A vaccine exposure requirement was added [17].
- Case definition changes: An updated version of the case definition was used.
- Statistical noise from the small sample size: The 95% CI positive predictive value (PPV) for this study was 39.6%, between 37.3% and 76.9%, suggesting statistical noise. A 2-sample proportion test comparing PPVs for the 2 studies is significant at a *P*<.01. However, the number of cases with insufficient evidence complicates comparing algorithm PPVs. Due to the rarity of postvaccination myocarditis/pericarditis, we included all the cases identified by our algorithm at site #2.
- Different levels of evidence: Categorizing cases with insufficient evidence was challenging in both studies. We assumed that the cases from the PPV were as likely to be positive or negative as unreviewed cases, with missing details possibly not documented for various reasons. We excluded these cases from the PPV calculation. However, it is possible that the lack of evidence indicated a clinician's doubt about the diagnosis, leading to less detailed notes or fewer diagnostic tests. This could vary across sites due to differences in processes, patient populations, and available technology. Notably, the previous validation excluded a quarter of myocarditis cases due to insufficient evidence, compared to less than 8% in this study.

### Methodological Considerations and Limitations

There were several limitations to this study. First, there were issues with measuring and comparing the PPV to our previous site (site #1) validation, as discussed above, including small sample sizes and challenges in handling insufficient evidence cases. Second, although the algorithms were designed to be simple to employ, they required customization from CQL or observational medical outcomes partnership queries to be implemented in the existing EHR system's available querying tools. Additionally, a substantial amount of time was needed to design and optimize the queries for large EHR databases. This customization must be made easier for health care data partners in the future.

We collected feedback from our data partner regarding potential concerns and improvements to make it easier to apply the algorithm to another site without sacrificing accuracy. Our data partner suggested that the query implementation time could be drastically reduced if we provided a design template for efficient phenotype queries and validations specific to an EHR vendor’s system; added additional text description around some of the phenotype logic; and simplified the algorithms to make them easier to implement.

Future research involving a new partner site can be designed to assess the reduction in time with the template from site #2, along with simplified and improved algorithm documentation.

Even with the recommended improvements, the customization required makes scaling the algorithms nationwide challenging. In the future, the evolving landscape of health IT may facilitate the public health use cases of detecting and reporting postvaccination AESIs in a safe and secure manner that protects patient privacy over the process described in this paper.

A truly interoperable ability to query across health providers is a demonstrated need to address variations in underlying technology, standards, and connectivity. The stakeholders include the electronic clinical report program jointly managed by the Association of Public Health Laboratories, Centers for Disease Control and Prevention, and Council of State and Territorial Epidemiologists; the Electronic medical record Support for Public health network for case reporting [18]; and the Helios HL7 FHIR Accelerator, Aggregate Data initiative [19], which has recently focused on capacity reporting such as open beds, ICU beds, emergency department visits, and similar statistics supporting emerging public health responses.

These initiatives vary in the complexity of the queries or trigger codes that identify a data point. A common thread is the inclusion or centrality of FHIR standards or profiles. A challenge for the BEST use case is the need for more complex queries linking biologic exposures to potential outcomes. This could be achieved by EHRs supporting secure querying of patient cohorts with probable postvaccination AEs using CQL [20] or another interoperable query language. Reducing the burden of automatic detection of postvaccination AEs would help public health organizations improve AE surveillance with minimal additional burden to health care institutions and providers.

A final limitation of this study is that the algorithms were only applied to a limited number of sites. Moving forward, the algorithm’s performance needs to be validated through exchanges at additional sites, preferably with a larger sample of potential postvaccination AE cases. This would ensure the following (Textbox 2).

**Algorithm generalizability and challenges in implementation across diverse healthcare settings and electronic health record systems.**

- Performance generalizes to other health care sites: The algorithm has only been applied to site #1 and site #2. It may have different operating characteristics (positive predictive value, sensitivity, etc) at additional locations.
- Performance generalizes to other postvaccination adverse events (AEs): Other algorithms could not be validated due to sample size issues arising from the rarity of postvaccination AEs. These algorithms may be validated with a larger data partner or a series of validations at different data partners.
- Algorithms can be applied to other sites’ electronic health record (EHR) implementations: Although the phenotype was applied to one Epic site, the results could differ for other Epic EHR implementations. We are currently researching variability in Fast Healthcare Interoperability Resources (FHIR) end points and how it might affect postmarket vaccine safety and effectiveness research (Deady et al, in preparation).
- Data received from other EHR vendor systems are similarly robust: Data captured or received through the exchange platform could vary among health care providers using other vendors’ EHR systems, altering the validation results and reducing the usefulness of this method for postvaccination AE detection.

### Conclusions

Active surveillance systems have the potential to enhance vaccine safety, contribute to the development and use of safer vaccines, and provide evidence to minimize the risk of postvaccination AEs [21]. These systems can also detect suspected or unknown AE cases. The algorithms described herein were developed and validated using a methodology that is recommended to be applied to and generalized for new EHR databases.

Before expanding the application of these algorithms, additional research is needed to improve their accuracy. This requires collaboration with additional partner EHR systems. Machine learning techniques could potentially be used to train the model to identify specific data patterns, creating a more effective algorithm compared to the rules-based methods that incorporate published case definition criteria and clinical subject matter experts. With sufficient data, machine learning approaches outperform rules-based approaches across domains, including the medical domain in which we received direct data extracts from EHR tables from our health care partner from site #1 [22].

However, machine learning methods may not be generalizable across different EHR systems because the data identified could be specific to individual health care organizations. Building a large dataset that combines multi-site data is challenging and costly due to concerns over infrastructure, regulations, privacy, and data standardization. Federated learning could be explored to address this problem. This method allows multiple sites to collaborate in training a global model without directly sharing data and has been used to train machine learning algorithms at EHR sites [23]. However, given the BEST initiatives’ goal of reducing burden on providers, federated learning faces multiple barriers to becoming nationally feasible. In the meantime, current methods of conducting large-scale surveillance studies with health care provider networks remain critical for postmarket safety and surveillance [24].

BEST staff will use the findings from this validation study to inform the long-term feasibility of scaling to a nationwide, semi-automated detection approach for an active surveillance system. Further research and investigation are needed to enhance algorithm performance and integration across health care organizations for active surveillance in the interest of public health.

## Appendix

Multimedia Appendix 1 Tables S1-S6 provide myocarditis and pericarditis case definition description, reference, computable phenotype search terms and code lists. Figure S1-S7 provide screenshots of the BEST Platform validation processes.

## Abbreviations

AE: adverse event
AESI: adverse event of special interest
BEST: Biologics Effectiveness and Safety
CBER: Center for Biologics Evaluation and Research
CONSORT: Consolidated Standards of Reporting Trials
CQL: clinical quality language
EHR: electronic health record
eHX: eHealth Exchange
FDA: US Food and Drug Administration
FHIR: Fast Healthcare Interoperability Resources
HL7: Health Level Seven, Inc
PPV: positive predictive value
RWD: real-world data
VAERS: vaccine adverse event reporting system
