# Supplementary material for: A Computable Phenotype Algorithm for Postvaccination Myocarditis/Pericarditis Detection Using Real-World Data: Validation Study
Source: J Med Internet Res. 2024 Nov 25;26:e54597. doi: 10.2196/54597 (PMC11629037; doi:10.2196/54597)
Supplement: Multimedia Appendix 1 [file jmir_v26i1e54597_app1.docx]

**Validation of a Computable Phenotype for Myocarditis/Pericarditis following COVID-19 Vaccinations Using a Pilot Active Surveillance Electronic Healthcare Data Exchange Platform** *Multimedia Appendix*

**Document Description**

This is a Multimedia Appendix to a full manuscript published in the J Med Internet Res. For full copyright and citation information see <https://www.jmir.org/2024/1/e54597>.

**Table S1: Myocarditis and pericarditis case definition description and reference**

| **AESI** | **Description** | **Case Definition Reference** |
| --- | --- | --- |
| **Myocarditis and Pericarditis** | Myocarditis and pericarditis are a spectrum of illnesses and frequently occur in combination. If symptoms of both exist, the level of certainty determination may differ for each diagnosis. Clinicians chose the higher of the two levels of certainty to make a diagnosis.  Findings in the following categories determine acute myocarditis and pericarditis:   - Signs and symptoms for myocarditis include dyspnea, palpitations, and chest pain of probable cardiac origin. For pericarditis, they include typical chest pain (worsened by lying down and relieved by sitting up or leaning forward), pleuritic chest pain, or peri-cardinal rub. - Histopathology evidence of myocardial or pericardial inflammation is required, along with positive signs/symptoms for all definite determinations. For probable or possible cases, histopathology evidence isn't required. Definite determinations in the presence of positive histopathology and signs/symptoms do not require further supporting EKG, imaging, or cardiac enzyme evidence. - Cardiac enzymes are evaluated only for myocarditis determinations. For probable cases, they must exhibit elevated troponin I or T or creatine kinase–myocardial band. Definite and possible cases do not require troponin evidence. - Electrocardiogram is helpful for distinguishing possible myocarditis cases, requiring ST-segment or T-wave abnormalities, arrhythmias, or AV nodal delays. Definite or probable myocarditis determinations do not require EKG changes. For pericarditis, only probable cases require EKG changes, including diffuse ST-segment elevations or PR depressions without reciprocal ST depressions. - Imaging studies: For myocarditis probable determinations, imaging should show evidence of depressed LV function, new or increased, or myocardial inflammation. For pericarditis probable determinations, echocardiogram should show an abnormal collection of pericardial fluid. | Morgan 2008[[1](#_ENREF_1)] |

**Table S2: Myocarditis / pericarditis search term appendix**

| **Data Element** | **Concept** | **Search Terms** |
| --- | --- | --- |
| Immunizations | Covid-19 | Covid-19 vaccine, SARS-COV-2 vaccine |
| Diagnosis | Myocarditis | Myocarditis |
|  | Pericarditis | Pericarditis |
| Labs & Observations | Creatine kinase | creatine, kinase |
|  | Ejection Fraction | ejection, fraction |
|  | Myocardial band | sedimentation, reactive, protein, complete, blood, count |
|  | Troponin | troponin |
|  | ACE Inhibitors | lisinopril, benazepril, enalapril, ramipril, quinapril, fosinopril, captopril, peridopril, trandolapril, moexipril, imidapril, enalaprilat, cilazapril, zofenopril, spirapril, delapril hydrochloride, temocapril hydrochloride |
| Medication | Anti-Inflammatory | NDC list |
|  | ARBs | losartan, valsartan, olmesartan, irbesartan, talmisartan, candesartan, azilsartan, eprosartan |
|  | Beta-adrenergic blockers | metoprolol, atenolol, carvedilol, popanolol, timolol, bisoprolol, nebivolol, labetalol, sotalol, nadolol, acebutolol, betaxolol, esmolol, pindolol, carteolol, celiprolol, oxprenolol, penbutolol, talinolol, tertatolol, alprenolol, bopindolol, bupranolol, mepindolol, practolol |
|  | Phosphodiesterase inhibitors | milrinone, inamrinone, enoximone, buclasdine sodium |
|  | Vasodilators | isosorbide, nitrolycerin, isosorbide dinitrate, nicorandil, heptaminol, molsidomine, pentaerythritol, nesiritide, trapidil, erythryityl tetranitrate, flosequinan, oxyfedrine, hexobendine, prenylamine, efloxate, chromonar |
| Procedure | Electrocardiogram | Electrocardiogram |

Abbreviations: ARBs, angiotensin receptor blockers; NDC, national drug code

**Table S3: Myocarditis / Pericarditis diagnosis codes**

| **Coding System** | **Code** | **Display** |
| --- | --- | --- |
| ICD-10-CM | I40.0 | Infective myocarditis |
| ICD-10-CM | I40.1 | Isolated myocarditis |
| ICD-10-CM | I40.8 | Other acute myocarditis |
| ICD-10-CM | I40.9 | Acute myocarditis, unspecified |
| ICD-10-CM | I51.4 | Viral myocarditis |
| ICD-10-CM | B33.22 | Viral pericarditis |
| ICD-10-CM | B33.23 | Acute rheumatic pericarditis |
| ICD-10-CM | I30.0 | Acute nonspecific idiopathic pericarditis |
| ICD-10-CM | I30.1 | Infective pericarditis |
| ICD-10-CM | I30.8 | Other forms of acute pericarditis |
| ICD-10-CM | I30.9 | Acute pericarditis, unspecified |
| ICD-10-CM | I32 | Pericarditis in diseases classified elsewhere |
| ICD-10-CM | I41 | Meningococcal pericarditis |
| SNOMED | 22653005 | Myocarditis due to infectious agent |
| SNOMED | 266238009 | Isolated (Fiedler's) myocarditis |
| SNOMED | 46701001 | Acute myocarditis |
| SNOMED | 50920009 | Myocarditis |
| SNOMED | 89141000 | Viral myocarditis |
| SNOMED | 70189005 | Viral pericarditis |
| SNOMED | 266235007 | Acute idiopathic pericarditis |
| SNOMED | 41739008 | Infectious pericarditis |
| SNOMED | 15555002 | Acute pericarditis |
| SNOMED | 3238004 | Pericarditis |
| SNOMED | 50920009 | Myocarditis (disorder) |

Abbreviations: ICD-10-CM, International Classification of Diseases, 10th Revision, clinical modification; SNOMED, Systematized Nomenclature of Medicine

**Table S4: Covid immunization codes**

| **Coding System** | **Code** | **Display** |
| --- | --- | --- |
| CVX | 207 | COVID-19, mRNA, LNP-S, PF, 100 mcg/0.5 mL dose |
| CVX | 208 | COVID-19, mRNA, LNP-S, PF, 30 mcg/0.3 mL dose |
| CVX | 212 | COVID-19 vaccine, vector-nr, rS-Ad26, PF, 0.5 mL |
| CVX | 213 | SARS-COV-2 (COVID-19) vaccine, UNSPECIFIED |
| CVX | 217 | SARS-COV-2 (COVID-19) vaccine, mRNA, spike protein, LNP, preservative free, 30 mcg/0.3mL dose, tris-sucrose formulation for 12 years and older |
| CVX | 218 | SARS-COV-2 (COVID-19) vaccine, mRNA, spike protein, LNP, preservative free, 10 mcg/0.2mL dose, tris-sucrose formulation; for 5-11 years |
| CVX | 219 | SARS-COV-2 (COVID-19) vaccine, mRNA, spike protein, LNP, preservative free, 3 mcg/0.2mL dose, tris-sucrose formulation; for 2-4 years |
| RxNorm | 2470234 | SARS-CoV-2 (COVID-19) vaccine, mRNA-1273 0.2 MG/ML Injectable Suspension |
| RxNorm | 2583743 | SARS-CoV-2 (COVID-19) vaccine, mRNA-BNT162b2 0.05 MG/ML Injectable Suspension |
| RxNorm | 2468235 | SARS-CoV-2 (COVID-19) vaccine, mRNA-BNT162b2 0.1 MG/ML Injectable Suspension |
| RxNorm | 2479835 | SARS-COV-2 (COVID-19) vaccine, vector - Ad26 100000000000 UNT/ML Injectable Suspension |
| NDC | 59267-1000-01 | Severe acute respiratory syndrome coronavirus2 (SARS-CoV-2) (coronavirusdisease [COVID-19]) vaccine, mRNA-LNP, spike protein, preservative free, 30 mcg/0.3mL dosage, diluent reconstituted, for intramuscular use |
| NDC | 59267100001 | Severe acute respiratory syndrome coronavirus2 (SARS-CoV-2) (coronavirusdisease [COVID-19]) vaccine, mRNA-LNP, spike protein, preservative free, 30 mcg/0.3mL dosage, diluent reconstituted, for intramuscular use |
| NDC | 59267-1000-02 | Severe acute respiratory syndrome coronavirus2 (SARS-CoV-2) (coronavirusdisease [COVID-19]) vaccine, mRNA-LNP, spike protein, preservative free, 30 mcg/0.3mL dosage, diluent reconstituted, for intramuscular use |
| NDC | 59267100002 | Severe acute respiratory syndrome coronavirus2 (SARS-CoV-2) (coronavirusdisease [COVID-19]) vaccine, mRNA-LNP, spike protein, preservative free, 30 mcg/0.3mL dosage, diluent reconstituted, for intramuscular use |
| NDC | 59267-1000-03 | Severe acute respiratory syndrome coronavirus2 (SARS-CoV-2) (coronavirusdisease [COVID-19]) vaccine, mRNA-LNP, spike protein, preservative free, 30 mcg/0.3mL dosage, diluent reconstituted, for intramuscular use |
| NDC | 59267100003 | Severe acute respiratory syndrome coronavirus2 (SARS-CoV-2) (coronavirusdisease [COVID-19]) vaccine, mRNA-LNP, spike protein, preservative free, 30 mcg/0.3mL dosage, diluent reconstituted, for intramuscular use |
| NDC | 0069-1000-03 | Severe acute respiratory syndrome coronavirus2 (SARS-CoV-2) (coronavirusdisease [COVID-19]) vaccine, mRNA-LNP, spike protein, preservative free, 30 mcg/0.3mL dosage, diluent reconstituted, for intramuscular use |
| NDC | 0069100003 | Severe acute respiratory syndrome coronavirus2 (SARS-CoV-2) (coronavirusdisease [COVID-19]) vaccine, mRNA-LNP, spike protein, preservative free, 30 mcg/0.3mL dosage, diluent reconstituted, for intramuscular use |
| NDC | 0069-1000-02 | Severe acute respiratory syndrome coronavirus2 (SARS-CoV-2) (coronavirusdisease [COVID-19]) vaccine, mRNA-LNP, spike protein, preservative free, 30 mcg/0.3mL dosage, diluent reconstituted, for intramuscular use |
| NDC | 0069100002 | Severe acute respiratory syndrome coronavirus2 (SARS-CoV-2) (coronavirusdisease [COVID-19]) vaccine, mRNA-LNP, spike protein, preservative free, 30 mcg/0.3mL dosage, diluent reconstituted, for intramuscular use |
| NDC | 59267-0078-01 | Severe acute respiratory syndrome coronavirus 2 (SARS-CoV-2) (coronavirus disease [COVID-19]) vaccine, mRNA-LNP, spike protein, preservative free, 30 mcg/0.3 mL dosage, tris-sucrose formulation, for intramuscular use; third dose |
| NDC | 59267007801 | Severe acute respiratory syndrome coronavirus 2 (SARS-CoV-2) (coronavirus disease [COVID-19]) vaccine, mRNA-LNP, spike protein, preservative free, 30 mcg/0.3 mL dosage, tris-sucrose formulation, for intramuscular use; third dose |
| NDC | 59267-0078-02 | Severe acute respiratory syndrome coronavirus 2 (SARS-CoV-2) (coronavirus disease [COVID-19]) vaccine, mRNA-LNP, spike protein, preservative free, 30 mcg/0.3 mL dosage, tris-sucrose formulation, for intramuscular use; third dose |
| NDC | 59267007802 | Severe acute respiratory syndrome coronavirus 2 (SARS-CoV-2) (coronavirus disease [COVID-19]) vaccine, mRNA-LNP, spike protein, preservative free, 30 mcg/0.3 mL dosage, tris-sucrose formulation, for intramuscular use; third dose |
| NDC | 59267-0078-04 | Severe acute respiratory syndrome coronavirus 2 (SARS-CoV-2) (coronavirus disease [COVID-19]) vaccine, mRNA-LNP, spike protein, preservative free, 30 mcg/0.3 mL dosage, tris-sucrose formulation, for intramuscular use; third dose |
| NDC | 59267007804 | Severe acute respiratory syndrome coronavirus 2 (SARS-CoV-2) (coronavirus disease [COVID-19]) vaccine, mRNA-LNP, spike protein, preservative free, 30 mcg/0.3 mL dosage, tris-sucrose formulation, for intramuscular use; third dose |
| NDC | 59267-1025-01 | Severe acute respiratory syndrome coronavirus 2 (SARS-CoV-2) (coronavirus disease [COVID-19]) vaccine, mRNA-LNP, spike protein, preservative free, 30 mcg/0.3 mL dosage, tris-sucrose formulation, for intramuscular use; third dose |
| NDC | 59267102501 | Severe acute respiratory syndrome coronavirus 2 (SARS-CoV-2) (coronavirus disease [COVID-19]) vaccine, mRNA-LNP, spike protein, preservative free, 30 mcg/0.3 mL dosage, tris-sucrose formulation, for intramuscular use; third dose |
| NDC | 59267-1025-03 | Severe acute respiratory syndrome coronavirus 2 (SARS-CoV-2) (coronavirus disease [COVID-19]) vaccine, mRNA-LNP, spike protein, preservative free, 30 mcg/0.3 mL dosage, tris-sucrose formulation, for intramuscular use; third dose |
| NDC | 59267102503 | Severe acute respiratory syndrome coronavirus 2 (SARS-CoV-2) (coronavirus disease [COVID-19]) vaccine, mRNA-LNP, spike protein, preservative free, 30 mcg/0.3 mL dosage, tris-sucrose formulation, for intramuscular use; third dose |
| NDC | 59267-1025-04 | Severe acute respiratory syndrome coronavirus 2 (SARS-CoV-2) (coronavirus disease [COVID-19]) vaccine, mRNA-LNP, spike protein, preservative free, 30 mcg/0.3 mL dosage, tris-sucrose formulation, for intramuscular use; third dose |
| NDC | 59267102504 | Severe acute respiratory syndrome coronavirus 2 (SARS-CoV-2) (coronavirus disease [COVID-19]) vaccine, mRNA-LNP, spike protein, preservative free, 30 mcg/0.3 mL dosage, tris-sucrose formulation, for intramuscular use; third dose |
| NDC | 59267-1055-01 | COVID-19, mRNA, LNP-S, PF, 10 mcg/0.2 mL dose, tris-sucrose |
| NDC | 59267105504 | COVID-19, mRNA, LNP-S, PF, 10 mcg/0.2 mL dose, tris-sucrose |
| NDC | 59267-1055-04 | COVID-19, mRNA, LNP-S, PF, 10 mcg/0.2 mL dose, tris-sucrose |
| NDC | 59267105501 | COVID-19, mRNA, LNP-S, PF, 10 mcg/0.2 mL dose, tris-sucrose |
| NDC | 80777-0273-15 | MODERNA COVID-19 VACCINE- cx-024414 injection, suspension |
| NDC | 80777027315 | MODERNA COVID-19 VACCINE- cx-024414 injection, suspension |
| NDC | 80777-0273-98 | MODERNA COVID-19 VACCINE- cx-024414 injection, suspension |
| NDC | 80777027398 | MODERNA COVID-19 VACCINE- cx-024414 injection, suspension |
| NDC | 80777-0273-10 | MODERNA COVID-19 VACCINE- cx-024414 injection, suspension |
| NDC | 80777027310 | MODERNA COVID-19 VACCINE- cx-024414 injection, suspension |
| NDC | 80777-0273-99 | MODERNA COVID-19 VACCINE- cx-024414 injection, suspension |
| NDC | 80777027399 | MODERNA COVID-19 VACCINE- cx-024414 injection, suspension |
| NDC | 59676-0580-05 | Janssen COVID-19 Vaccine |
| NDC | 59676058005 | Janssen COVID-19 Vaccine |
| NDC | 59676-0580-15 | Janssen COVID-19 Vaccine |
| NDC | 59676058015 | Janssen COVID-19 Vaccine |

Abbreviations: NDC- national drug code; CVX- vaccine administered codes; RxNorm; normalized naming system for generic and branded drugs; LNP, lipid nanoparticle; PF, pulmonary fibrosis

**Table S5: Myocarditis / Pericarditis Observation Lab Test Codes**

| **System** | **Code** | **Display** |
| --- | --- | --- |
| LOINC | 48425-3 | Troponin T.cardiac [Mass/volume] in Blood |
| LOINC | 33204-9 | Troponin T.cardiac [Presence] in Serum or Plasma |
| LOINC | 10839-9 | Troponin I.cardiac [Mass/volume] in Serum or Plasma |
| LOINC | 16255-2 | Troponin I.cardiac [Units/volume] in Serum or Plasma |
| LOINC | 42757-5 | Troponin I.cardiac [Mass/volume] in Blood |
| LOINC | 48426-1 | Troponin T.cardiac [Presence] in Blood |
| LOINC | 49563-0 | Troponin I.cardiac [Mass/volume] in Serum or Plasma by Detection limit <= 0.01 ng/mL |
| LOINC | 89575-5 | Troponin T.cardiac [Interpretation] in Serum or Plasma Qualitative by High sensitivity method |
| LOINC | 6597-9 | Troponin T.cardiac [Mass/volume] in Venous blood |
| LOINC | 6598-7 | Troponin T.cardiac [Mass/volume] in Serum or Plasma |
| LOINC | 76399-5 | Troponin I.cardiac [Presence] in Serum, Plasma or Blood by Rapid immunoassay |
| LOINC | 67151-1 | Troponin T.cardiac [Mass/volume] in Serum or Plasma by High sensitivity method |
| LOINC | 89576-3 | Troponin T.cardiac panel - Serum or Plasma by High sensitivity method |
| LOINC | 89577-1 | Troponin I.cardiac panel - Serum or Plasma by High sensitivity method |
| LOINC | 89578-9 | Troponin I.cardiac [Interpretation] in Serum or Plasma Qualitative by High sensitivity method |
| LOINC | 89579-7 | Troponin I.cardiac [Mass/volume] in Serum or Plasma by High sensitivity method |
| LOINC | 13969-1 | Creatine kinase.MB [Mass/volume] in Serum or Plasma |
| LOINC | 12187-1 | Creatine kinase.MB/Creatine kinase.total in Serum or Plasma by Electrophoresis |
| LOINC | 12188-9 | Deprecated Creatine kinase.MB/Creatine kinase.total |
| LOINC | 12189-7 | Creatine kinase.MB/Creatine kinase.total in Serum or Plasma by calculation |
| LOINC | 15048-2 | Creatine kinase.BB/Creatine kinase.total in Serum or Plasma by Electrophoresis |
| LOINC | 14680-3 | Creatine kinase isoenzymes [Interpretation] in Serum or Plasma by Electrophoresis |
| LOINC | 15049-0 | Creatine kinase.MM/Creatine kinase.total in Serum or Plasma by Electrophoresis |
| LOINC | 2157-6 | Creatine kinase [Enzymatic activity/volume] in Serum or Plasma |
| LOINC | 2152-7 | Creatine kinase.BB [Enzymatic activity/volume] in Serum or Plasma by Electrophoresis |
| LOINC | 2153-5 | Creatine kinase.macromolecular [Enzymatic activity/volume] in Serum or Plasma |
| LOINC | 2154-3 | Creatine kinase.MB [Enzymatic activity/volume] in Serum or Plasma by Electrophoresis |
| LOINC | 2155-0 | Creatine kinase.MM [Enzymatic activity/volume] in Serum or Plasma by Electrophoresis |
| LOINC | 16688-4 | Creatine kinase [Enzymatic activity/volume] in Body fluid |
| LOINC | 20569-0 | Creatine kinase.MB/Creatine kinase.total in Serum or Plasma |
| LOINC | 2158-4 | Creatine kinase.total/Creatine kinase.MB [Enzymatic activity ratio] in Serum or Plasma |
| LOINC | 2156-8 | Creatine kinase [Enzymatic activity/volume] in Amniotic fluid |
| LOINC | 32673-6 | Creatine kinase.MB [Enzymatic activity/volume] in Serum or Plasma |
| LOINC | 2151-9 | Creatine kinase [Enzymatic activity/volume] in Cerebral spinal fluid |
| LOINC | 24335-2 | Creatine kinase panel - Serum or Plasma |
| LOINC | 26019-0 | Creatine Kinase.macromolecular type 1/Creatine kinase.total in Serum or Plasma |
| LOINC | 26020-8 | Creatine Kinase.macromolecular type 2/Creatine kinase.total in Serum or Plasma |
| LOINC | 34160-2 | Creatine kinase [Presence] in Body fluid |
| LOINC | 33547-1 | Creatine Kinase aberrant band/Creatine kinase.total in Serum or Plasma by Electrophoresis |
| LOINC | 38482-6 | Creatine kinase.MB [Presence] in Serum or Plasma |
| LOINC | 49258-7 | Creatine kinase isoenzymes [Interpretation] in Serum or Plasma by Electrophoresis Narrative |
| LOINC | 49129-0 | Creatine kinase.MiMi/Creatine kinase.total in Serum or Plasma |
| LOINC | 49136-5 | Creatine kinase.MB/Creatine kinase.total [Ratio] in Serum or Plasma |
| LOINC | 49259-5 | Creatine kinase isoenzymes [Interpretation] in Serum or Plasma Narrative |
| LOINC | 59149-5 | Creatine kinase.macromolecular [Presence] in Serum or Plasma |
| LOINC | 49551-5 | Creatine kinase.MB [Mass/volume] in Blood |
| LOINC | 53433-9 | Creatine kinase [Enzymatic activity/volume] in Dialysis fluid |
| LOINC | 50756-6 | Creatine kinase [Mass/volume] in Blood |
| LOINC | 50757-4 | Creatine kinase.total/Creatine kinase.MB [Enzymatic activity ratio] in Blood |
| LOINC | 51505-6 | Creatine kinase.BB [Enzymatic activity/volume] in Cerebral spinal fluid by Electrophoresis |
| LOINC | 51506-4 | Creatine kinase.MB [Enzymatic activity/volume] in Cerebral spinal fluid by Electrophoresis |
| LOINC | 51507-2 | Creatine kinase.MM [Enzymatic activity/volume] in Cerebral spinal fluid by Electrophoresis |
| LOINC | 5912-1 | Creatine kinase isoenzymes [Interpretation] in Serum or Plasma |
| LOINC | 9642-0 | Creatine kinase.BB/Creatine kinase.total in Serum or Plasma |
| LOINC | 6773-6 | Deprecated Creatine kinase.MB |
| LOINC | 72561-4 | Creatine Kinase aberrant band/Creatine kinase.total [Pure catalytic fraction] in Serum or Plasma by Electrophoresis |
| LOINC | 72562-2 | Creatine kinase.MM/Creatine kinase.total [Pure catalytic fraction] in Serum or Plasma by Electrophoresis |
| LOINC | 72563-0 | Creatine kinase.MB/Creatine kinase.total [Pure catalytic fraction] in Serum or Plasma by Electrophoresis |
| LOINC | 72564-8 | Creatine kinase.MB/Creatine kinase.total [Pure catalytic fraction] in Serum or Plasma by calculation |
| LOINC | 72565-5 | Creatine kinase.BB/Creatine kinase.total [Pure catalytic fraction] in Serum or Plasma by Electrophoresis |
| LOINC | 83092-7 | Creatine kinase.MB [Mass/volume] in Serum or Plasma by Immunoassay |
| LOINC | 9643-8 | Creatine kinase.MM/Creatine kinase.total in Serum or Plasma |
| LOINC | 11039-5 | C reactive protein [Presence] in Serum or Plasma |
| LOINC | 18184-2 | Erythrocyte sedimentation rate by 2H Westergren method |
| LOINC | 14634-0 | C reactive protein [Titer] in Serum or Plasma |
| LOINC | 16503-5 | C reactive protein [Mass/volume] in Body fluid |
| LOINC | 1988-5 | C reactive protein [Mass/volume] in Serum or Plasma |
| LOINC | 30341-2 | Erythrocyte sedimentation rate |
| LOINC | 30522-7 | C reactive protein [Mass/volume] in Serum or Plasma by High sensitivity method |
| LOINC | 35648-5 | C reactive protein [Quintile] in Serum or Plasma by High sensitivity method |
| LOINC | 43402-7 | Erythrocyte sedimentation rate by 15 minute reading |
| LOINC | 45062-7 | C reactive protein [Mass/volume] in Cerebral spinal fluid |
| LOINC | 93340-8 | PhenX - complete blood count protocol 220501 |
| LOINC | 76485-2 | C reactive protein [Moles/volume] in Serum or Plasma |
| LOINC | 76486-0 | C reactive protein [Moles/volume] in Serum or Plasma by High sensitivity method |
| LOINC | 82477-1 | Erythrocyte sedimentation rate by Photometric method |
| LOINC | 4537-7 | Erythrocyte sedimentation rate by Westergren method |
| LOINC | 4538-5 | Erythrocyte sedimentation rate by Wintrobe method |
| LOINC | 4539-3 | Erythrocyte sedimentation rate Zeta by Zetafuge |
| LOINC | 48421-2 | C reactive protein [Mass/volume] in Capillary blood |
| LOINC | 59182-6 | C reactive protein [Mass/volume] in Cerebral spinal fluid by High sensitivity method |
| LOINC | 71426-1 | C reactive protein [Mass/volume] in Blood by High sensitivity method |
| LOINC | 18043-0 | Left ventricular Ejection fraction by US |
| LOINC | 10231-9 | Right ventricular Ejection fraction |
| LOINC | 10230-1 | Left ventricular Ejection fraction |
| LOINC | 18044-8 | Left ventricular Ejection fraction by US.2D+Calculated by single-plane ellipse method |
| LOINC | 18045-5 | Left ventricular Ejection fraction by US.2D+Calculated by biplane ellipse method |
| LOINC | 18046-3 | Left ventricular Ejection fraction by US 2D modified |
| LOINC | 18047-1 | Left ventricular Ejection fraction by US 2D modified biplane |
| LOINC | 18048-9 | Left ventricular Ejection fraction by US 2D modified single-plane |
| LOINC | 18049-7 | Left ventricular Ejection fraction by US.M-mode+Calculated by Teichholz method |
| LOINC | 39887-5 | NM Heart First pass and Ejection fraction at rest and W radionuclide IV |
| LOINC | 24571-2 | NM Biliary ducts and Gallbladder Views for patency of biliary structures and ejection fraction W sincalide and W radionuclide IV |
| LOINC | 39889-1 | NM Heart First pass and Ejection fraction |
| LOINC | 39895-8 | NM Gallbladder Views for ejection fraction W Tc-99m DISIDA IV |
| LOINC | 81567-0 | NM Heart Gated and Blood pool and Ejection fraction and Wall motion W single state of exercise |
| LOINC | 46395-0 | SPECT Heart gated and ejection fraction at rest and W stress and W radionuclide IV |
| LOINC | 55406-3 | Left ventricular Cardiac ejection fraction method |
| LOINC | 81388-1 | Right ventricular Ejection fraction by US 3D |
| LOINC | 39909-7 | NM Heart First pass and Wall motion and Ventricular volume and Ejection fraction W stress and W radionuclide IV |
| LOINC | 39910-5 | NM Heart First pass and Wall motion and Ejection fraction |
| LOINC | 39911-3 | Deprecated Heart Scan first pass & wall motion & ejection fraction single view |
| LOINC | 39912-1 | NM Heart First pass and Wall motion and Ventricular volume and Ejection fraction |
| LOINC | 39913-9 | SPECT Heart gated and ejection fraction |
| LOINC | 39917-0 | NM Heart Gated and Ejection fraction |
| LOINC | 39923-8 | NM Heart Gated and Ejection fraction at rest and W radionuclide IV |
| LOINC | 39925-3 | NM Heart Gated and Wall motion and Ejection fraction at rest and W radionuclide IV |
| LOINC | 39931-1 | NM Heart Gated and Wall motion and Ejection fraction |
| LOINC | 39932-9 | NM Heart Wall motion and Ejection fraction |
| LOINC | 77889-4 | Left ventricular Ejection fraction by US.M-mode+Calculated by cube method |
| LOINC | 77890-2 | Left ventricular Ejection fraction by US.2D+Calculated by cube method |
| LOINC | 77891-0 | Left ventricular Ejection fraction by US.2D+Calculated by Teichholz method |
| LOINC | 77892-8 | Left ventricular Ejection fraction by US.2D+Calculated by modified Simpson method |
| LOINC | 79990-8 | Left ventricular Ejection fraction by US.3D.segmentation |
| LOINC | 79991-6 | Left ventricular Ejection fraction by US.2D+Calculated by biplane method of disks |
| LOINC | 79992-4 | Left ventricular Ejection fraction by US.2D.A2C+Calculated by single plane method of disks |
| LOINC | 79993-2 | Left ventricular Ejection fraction by US.2D.A4C+Calculated by single plane method of disks |
| LOINC | 81566-2 | NM Heart Gated and Blood pool and Ejection fraction and Wall motion W multiple states of exercise |
| LOINC | 81612-4 | NM Heart Blood pool and Ejection fraction and First pass |
| LOINC | 8809-6 | Left ventricular Ejection fraction by Cardiac angiogram.visual estimate |
| LOINC | 8806-2 | Left ventricular Ejection fraction by 2D echo |
| LOINC | 8810-4 | Left ventricular Ejection fraction by Spiral CT |
| LOINC | 8807-0 | Left ventricular Ejection fraction by 2D echo.visual estimate |
| LOINC | 8811-2 | Left ventricular Ejection fraction by MR |
| LOINC | 8814-6 | Right ventricular Ejection fraction by 2D echo.visual estimate |
| LOINC | 8812-0 | Left ventricular Ejection fraction by Nuclear blood pool |
| LOINC | 8815-3 | Right ventricular Ejection fraction by Cardiac angiogram |
| LOINC | 8808-8 | Left ventricular Ejection fraction by Cardiac angiogram |
| LOINC | 8818-7 | Right ventricular Ejection fraction by MR |
| LOINC | 8813-8 | Right ventricular Ejection fraction by 2D echo |
| LOINC | 8819-5 | Right ventricular Ejection fraction by Nuclear blood pool |
| LOINC | 8820-3 | Ventricular Ejection fraction geometry formula |
| LOINC | 8816-1 | Right ventricular Ejection fraction by Cardiac angiogram.visual estimate |
| LOINC | 93644-3 | Left ventricular Ejection fraction by US.2D.A2C+Calc by single plane area-length method |
| LOINC | 8817-9 | Right ventricular Ejection fraction by Spiral CT |
| LOINC | 93645-0 | Left ventricular Ejection fraction by US.2D.A4C+Calc by single plane area-length method |
| LOINC | 93646-8 | Left ventricular Ejection fraction by US.2D+Calculated by biplane area-length method |
| LOINC | 8814-6 | Right ventricular Ejection fraction by 2D echo.visual estimate |
| LOINC | 8812-0 | Left ventricular Ejection fraction by Nuclear blood pool |

Abbreviations: LOINC, Logical Observation Identifiers, Names, and Codes

**Table S6: Myocarditis / pericarditis medication code examples*******

| **System** | **Code** | **Display** |
| --- | --- | --- |
| RxNorm | 81947 | alprenolol hydrochloride |
| RxNorm | 94271 | Atenolol / Chlorthalidone Oral Tablet [Tenoretic] |
| RxNorm | 91991 | betaxolol Ophthalmic Solution [Betoptic] |
| RxNorm | 91990 | betaxolol Ophthalmic Suspension [Betoptic S] |
| RxNorm | 93053 | betaxolol Oral Tablet [Kerlone] |
| RxNorm | 19484 | bisoprolol |
| RxNorm | 19605 | bopindolol |
| RxNorm | 20498 | celiprolol |
| RxNorm | 49737 | esmolol |
| RxNorm | 92016 | esmolol Injectable Solution [Brevibloc] |
| RxNorm | 92962 | Hydrochlorothiazide / Propranolol Oral Tablet [inderide] |
| RxNorm | 29518 | mepindolol |
| RxNorm | 92254 | nadolol Oral Tablet [Corgard] |
| RxNorm | 31555 | nebivolol |
| RxNorm | 82070 | penbutolol sulfate |
| RxNorm | 92959 | Propranolol Extended Release Capsule [Inderal-LA] |
| RxNorm | 82084 | propranolol hydrochloride |
| RxNorm | 92958 | Propranolol Injectable Solution [Inderal] |
| RxNorm | 92960 | propranolol Oral Tablet [Inderal] |
| RxNorm | 37546 | talinolol |
| RxNorm | 37840 | tertatolol |
| RxNorm | 10600 | timolol |
| RxNorm | 42933 | timolol maleate |
| RxNorm | 92008 | timolol Oral Tablet [Blocadren] |

** Several thousand medications codes were used in this algorithm. Only a small sample of those that were used are included here. Please reach out to the authors for a full list of medication codes.*

Abbreviations: RxNorm, normalized naming system for generic and branded drugs

*
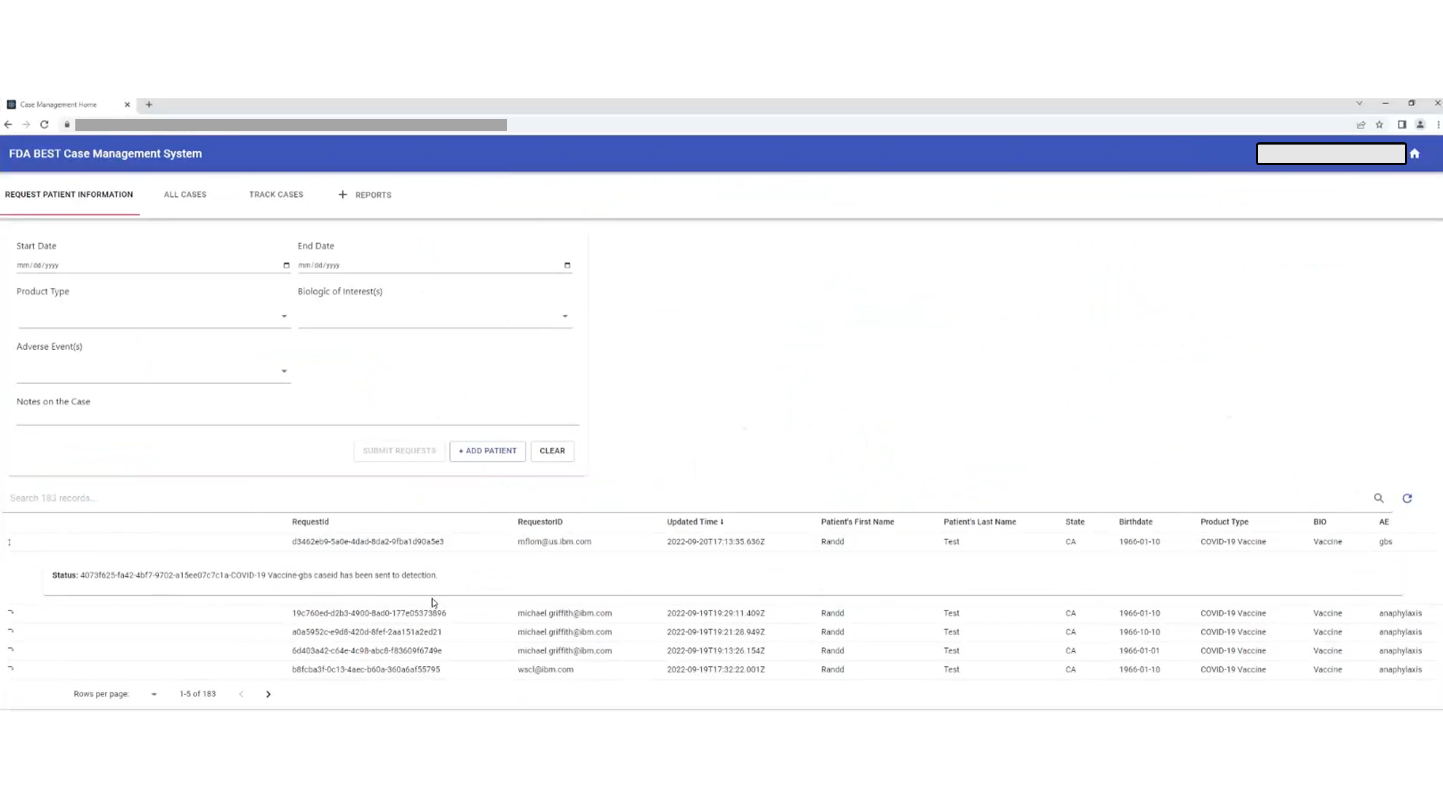
*

Figure S1. BEST Platform Case Management window, which allows the user to enter one, or more, patient(s) demographics; date of birth; suspected adverse event; start and end date of the relevant clinical data, and submit a query to eHealth Exchange. All data shown here are synthetic data used in the testing environment.

*
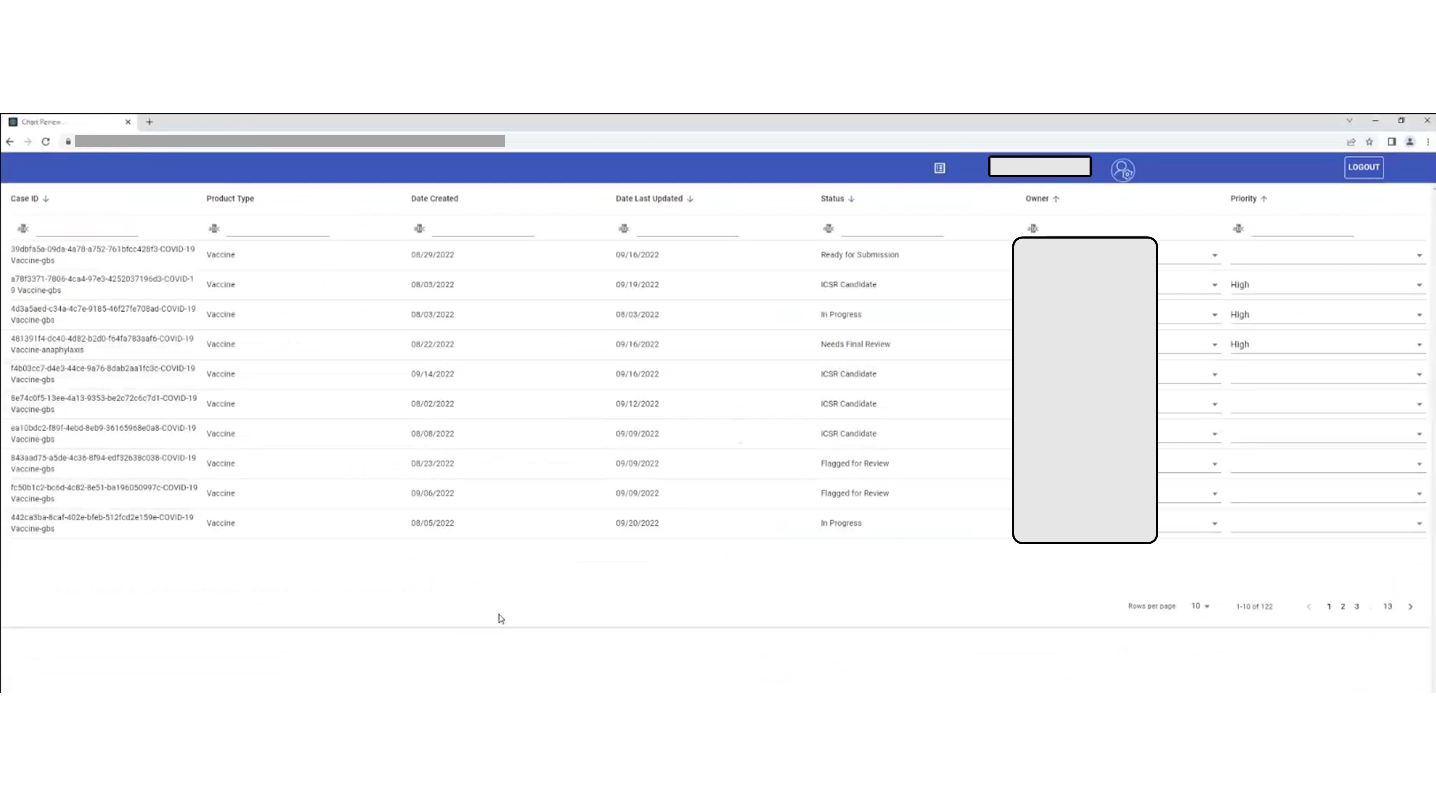
*

Figure S2. BEST Platform Case Management window displaying all queried and received cases.

*
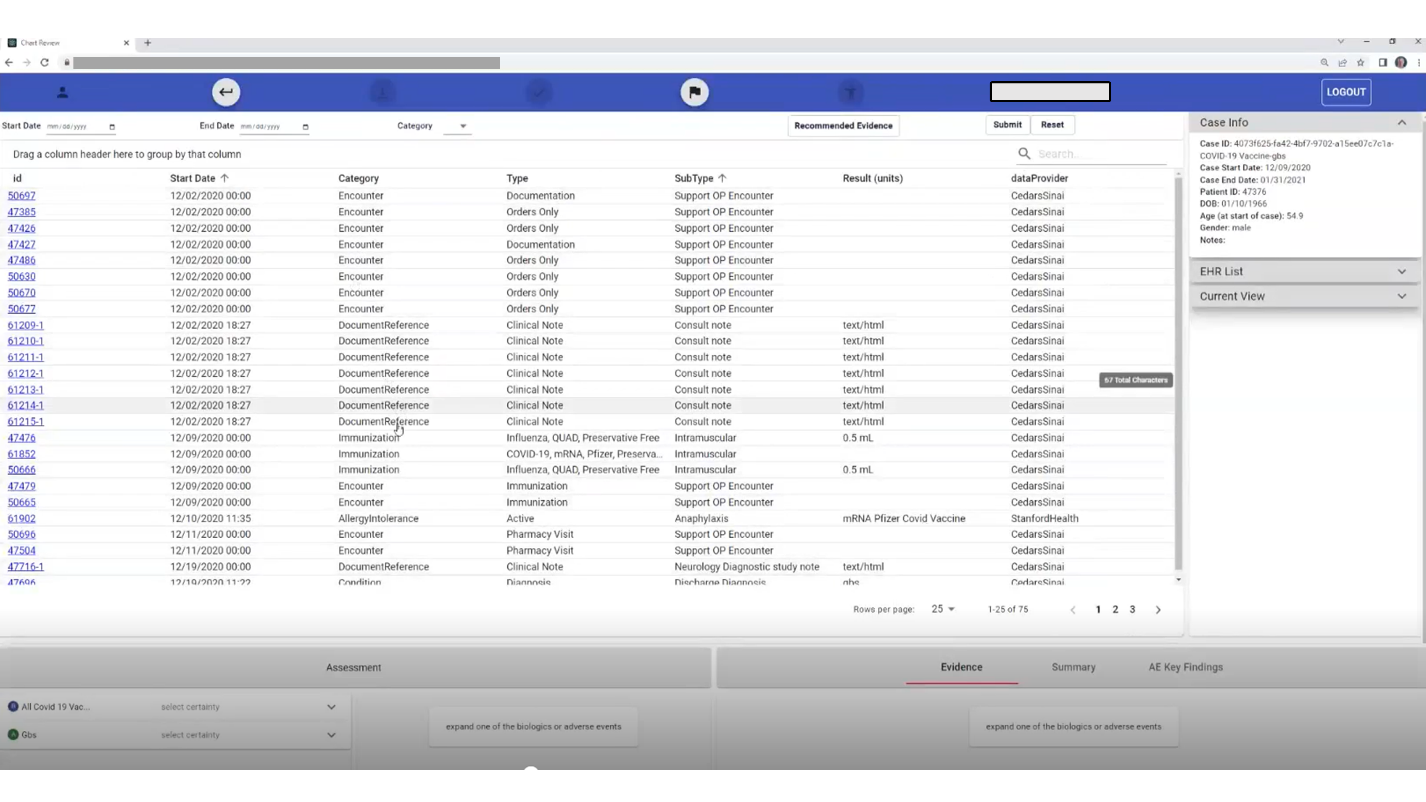
*

Figure S3. BEST Platform Chart review window. The top left part of the window shows the FHIR resources of a selected case. The top tight shows the case metadata. The bottom left part shows the “Assessment” section, where the clinician ascertains the exposure, the outcome, the severity, and the imputability. The bottom right part shows the results section, where the conclusion of the assessment is displayed with the information selected by the clinician as relevant to their assessment.

*
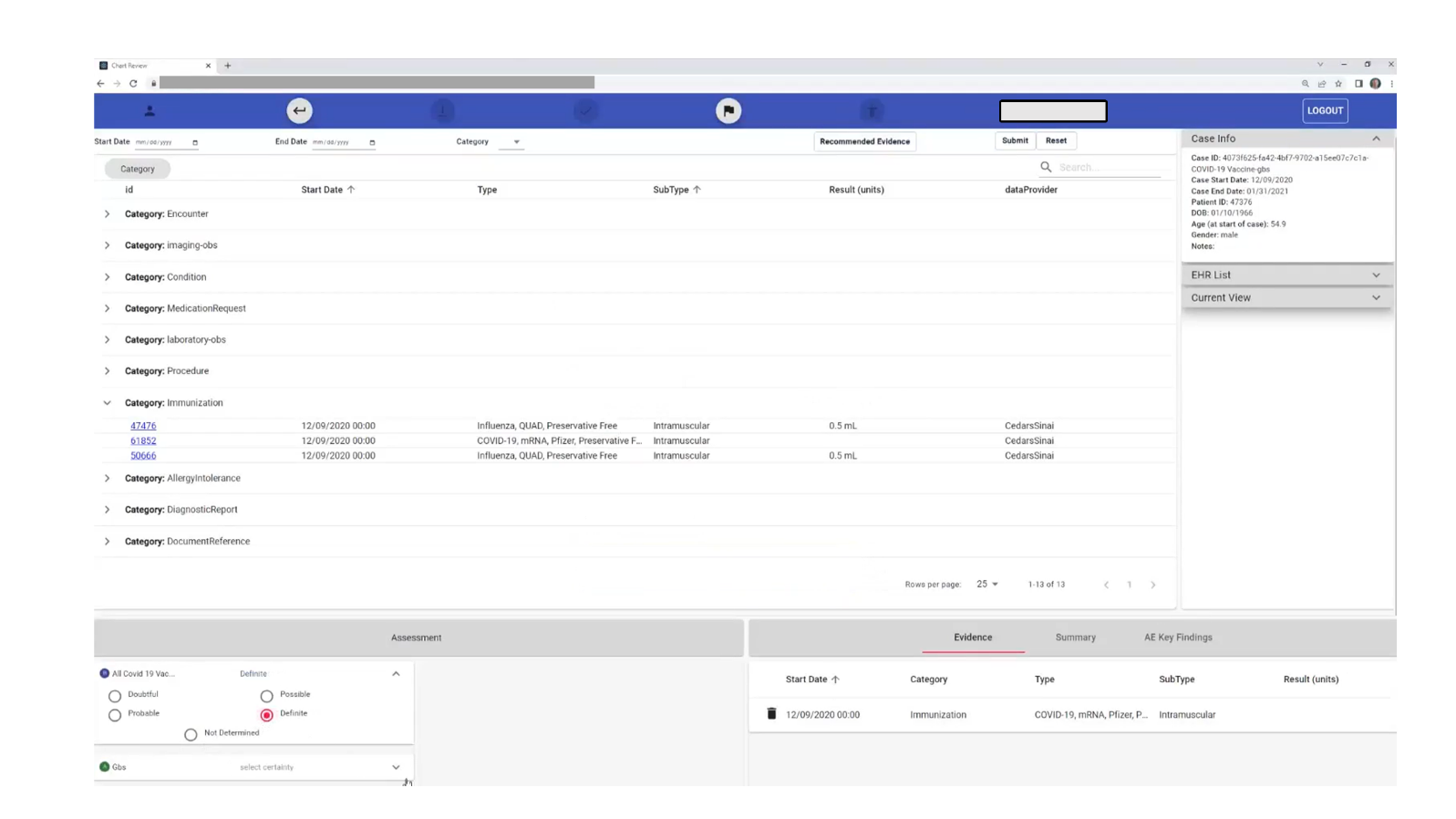
*

Figure S4. BEST Platform Chart review window showing the grouping feature, where the clinician is able to rearrange the FHIR resources based on different criteria. In this figure, “Category” in the criteria chosen to group the FHIR resources.

*
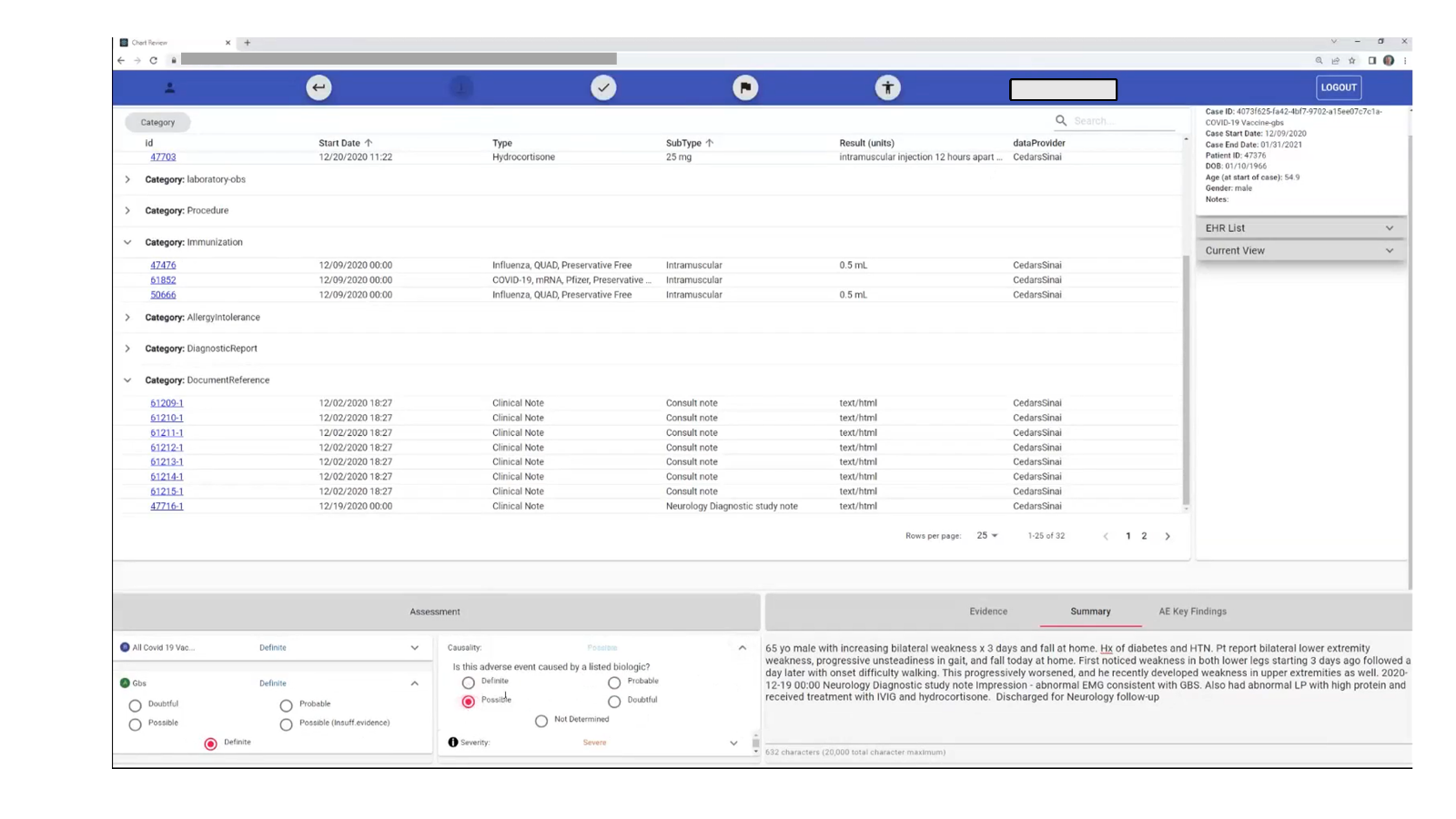
*

Figure S5. BEST Platform Chart Review window showing the clinician summary based on their assessment. The summary is then mapped to the narrative of the final case report (see Figure S7)

*
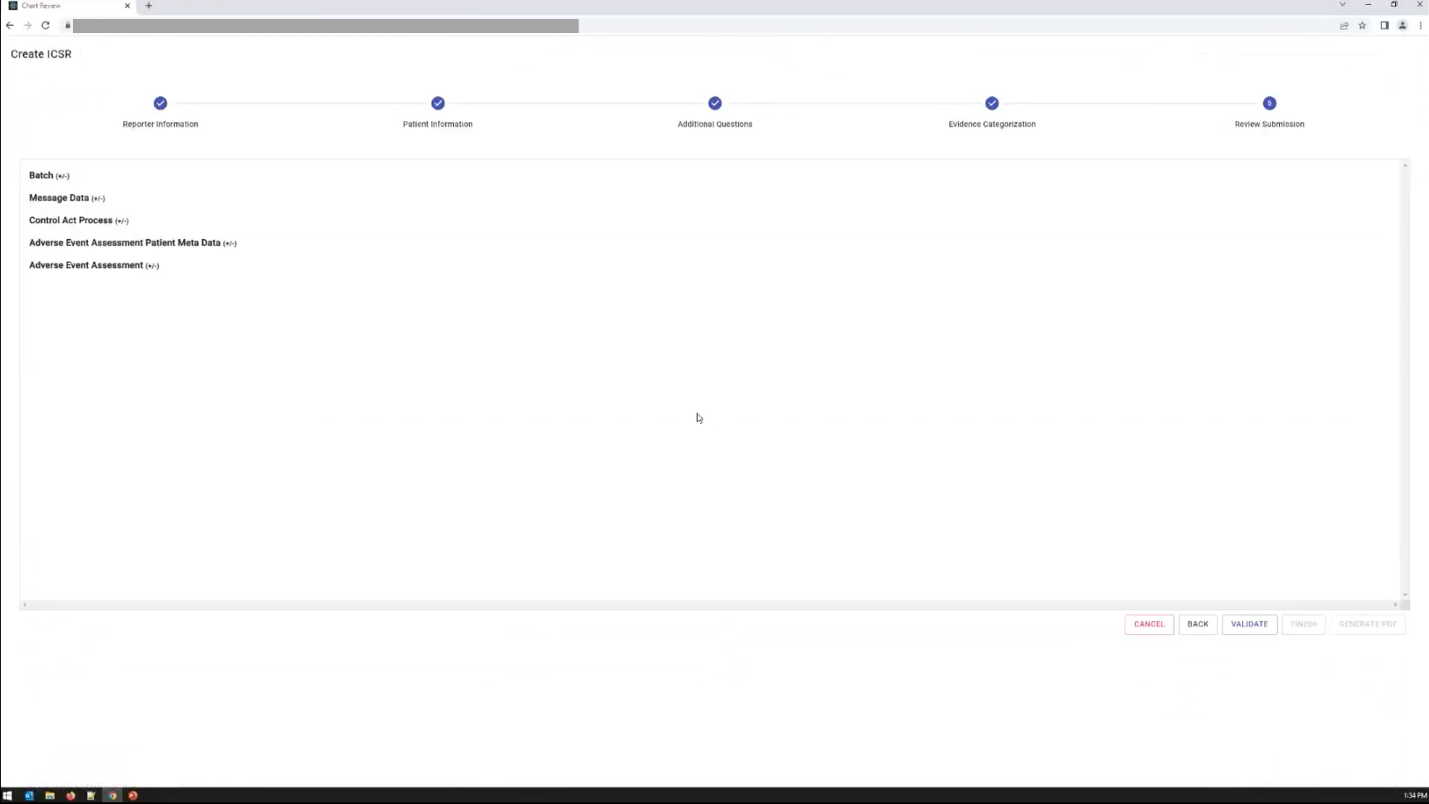
*

Figure S6. Report Validation window depicting the validation process, where the BEST Platform validates the mapped data elements and verifies the completeness of the required elements.

*
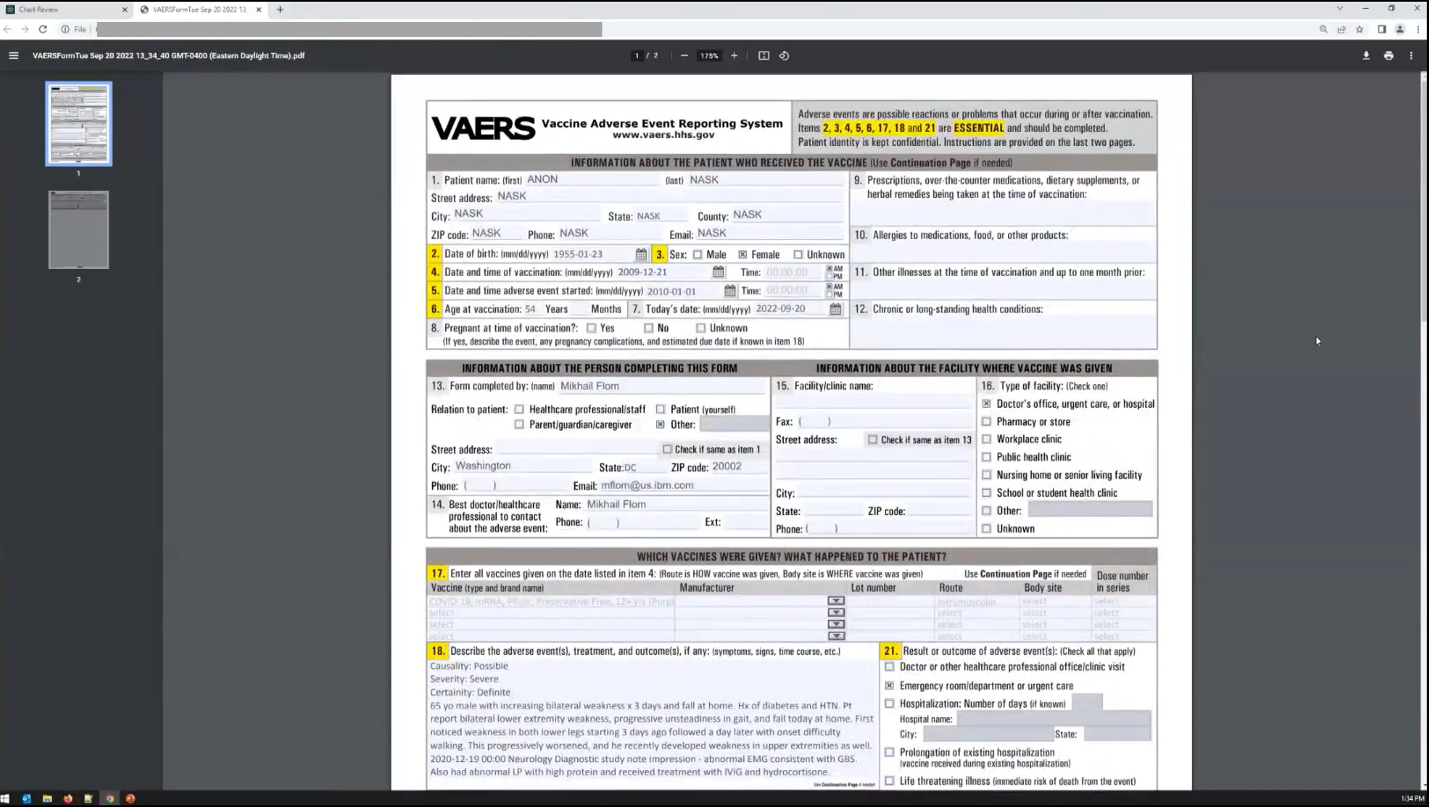
*

Figure S7 VAERS-like report generated by the BEST Platform based on the clinician review and validation of a case. The report is generated by mapping the received FHIR-based clinical data elements and the notes highlighted by the clinician into the corresponding VAERS report fields.

1. Morgan J, Roper MH, Sperling L, Schieber RA, Heffelfinger JD, Casey CG, et al. Myocarditis, pericarditis, and dilated cardiomyopathy after smallpox vaccination among civilians in the United States, January-October 2003. Clin Infect Dis. 2008.
